# A systematic review of adolescent physiological development and its relationship with health-related behaviour: a protocol

**DOI:** 10.1186/s13643-015-0173-5

**Published:** 2016-01-05

**Authors:** Jan Pringle, Kate Mills, John McAteer, Ruth Jepson, Emma Hogg, Neil Anand, Sarah-Jayne Blakemore

**Affiliations:** Scottish Collaboration for Public Health Research & Policy (SCPHRP), University of Edinburgh, 20 West Richmond St, Edinburgh, EH8 9DX UK; NHS Health Scotland, Edinburgh, UK; University College London, London, UK

## Abstract

**Background:**

At any one time, there are one billion people worldwide who are in the second decade of their life, and 1.8 billion in the 10–24 age range.

Whilst a great deal of focus has been placed on healthy early years development, the adolescent years are also a unique period of opportunity: exposure to health-influencing behaviours such as alcohol consumption or cigarette smoking, may serve to establish patterns that have significant health consequences in later life. Although there is often an emphasis on risk-taking and detrimental health behaviours during adolescence, these years also provide significant opportunities for behaviour to be shaped in positive ways that may improve longer term health outcomes. However, it is firstly important to understand the complex physiological changes that are taking place within the human body during this period and their relationship with health-related behaviour. Such knowledge can help to inform health policy and intervention development.

**Aim:**

The aim of this study is to gain a comprehensive understanding of the relationship between physiological development and health-related behaviours in adolescence.

**Methods:**

The principles of an integrative review will be used. Such reviews are of use where research has emerged in different fields, to combine existing knowledge and produce a more extensive understanding. Studies from a range of different methodological approaches, published or unpublished, will be included. A range of databases and literature depositories will be searched using a pre-defined search strategy. The review will include studies that focus on adolescents (nominally, those aged 10–24 years). We will seek papers that focus on both physiological development and health behaviour, or papers focusing solely on physiological development if there are clear implications for health behaviour. Studies with a focus on participants with specific health conditions will be excluded.

Two reviewers will independently screen potential studies for eligibility and quality; members of the project team will act as third reviewers in the case of uncertainty or discrepancy.

Further analyses (e.g. meta-analysis, meta-synthesis, meta-summary) will be decided upon, and sub-set analyses carried out. Finally, an integrative summation will be produced, giving a critical analysis of the results and providing conclusions and recommendations.

**Electronic supplementary material:**

The online version of this article (doi:10.1186/s13643-015-0173-5) contains supplementary material, which is available to authorized users.

## Introduction and background

At any one time, there are one billion people worldwide who are in the second decade of their life [1] and 1.8 billion in the 10–24 age range [2]. Broadly speaking, adolescence occurs in and around the second decade and is the transitional developmental period between childhood and adulthood. The term adolescence, derived from the latin meaning for ‘growing up’, has no universally accepted definition. For example, WHO [2] describes adolescence as a period in human growth and development that occurs nominally between the ages of 10 and 19. Others define it as incorporating young adulthood, spanning a variable period of time between the ages of 10 and 24 [3]. There is widespread agreement that adolescence starts at puberty and ends with the uptake of mature social roles, such as employment and child rearing. Due to the later transition into these roles in many developed countries, the longer and later definition may therefore be more appropriate. There are, however, recognised wide variations in the start and end points of adolescence, including differences between cultures and individuals [4].

Whilst a great deal of focus has been placed on the importance of healthy early childhood development [5–7], the adolescent years are a unique period in their own right. For example, during this period, there is increased exposure to health-influencing behaviours such as alcohol consumption and cigarette smoking. These behaviours, in conjunction with peer group pressure, may contribute to the establishment of patterns that have significant health consequences in later life [8]. However, whilst there is often an emphasis on risk and detrimental health behaviours during adolescence [9], these years also provide significant opportunities for behaviour to be shaped in positive ways that may improve longer term health outcomes [1]. The capacity of adolescents to make decisions and take control of their own future life course should not be underestimated, especially if they are appropriately informed.

Intervening to support positive health choices during adolescence therefore has the potential to lessen the likelihood of developing a health condition in later life, with possible positive implications for future health service provision and resources. An understanding of the complex physiological changes that are taking place within the human body during this period and the relationship with health-related behaviours is the first step towards recognising adolescence as a transition period that warrants a specific focus as a unique period of opportunity. Such knowledge can help to inform health policy and intervention development.

The aim of this systematic review is to gain an understanding of the relationship between physiological development and health-related behaviours in adolescence. The objectives are to:

1. Identify and describe the range of evidence that explores the relationship between physiological development and health behaviours in adolescence
2. Determine the strength of such evidence

This review forms the first phase of a wider adolescent and young adult research programme (see Additional file 1). It is hoped that the findings of the review will help to inform policy, practice, and future research priorities.

## Methods

Previous associated reviews [9, 10], which have both focussed on adolescent health in more general terms, indicate that data may be found from a broad range of sources. In order to encompass such a wide variety of literature, this systematic review will be based on the principles of an integrative review. This approach can be used to capture an extensive range of studies with a common focus on the chosen topic of interest [11]. Integrative reviews are the most comprehensive of all review approaches, facilitating the inclusion of different methodological approaches, and both published or unpublished literature [12]. Such reviews can combine existing knowledge to produce a more extensive understanding [13].

Within a systematic integrative review, similar studies can be grouped together (e.g. clinical trials and comparative studies or qualitative and descriptive studies). Quality criteria instruments and analytical methods can then be selected to be relevant to each type.

The stages of the review are summarised in Table 1, with key elements of the review being further detailed below.

**Table 1:** Five stages of an integrative literature review (summarised from Whittemore and Knafl [11])

Stages of review	Aim/purpose	Details
(1) Problem formulation	To clearly state topic of interest and purpose of review	• List variables of interest • Set focus and boundaries
(2) Literature search	To make explicit and justify search strategy and sampling criteria	• Specify databases and other search methods • State type of literature to be included (e.g. published/unpublished) • Detail key words • Acknowledge publication bias
(3) Data evaluation	To assess type, scope, diversity, and quality of accessed literature	• Specify different types of study found and classify into sub-groups • Decide on quality criteria instruments for each type of study
(4) Data synthesis	To specify systematic analytical method To create an innovative synthesis To formulate a unified and integrated conclusion	• Data reduction: simplify sub-groups into a manageable framework according to the type (e.g. qualitative, comparative, experimental); create short summaries of each primary source • Data display: create charts or visual network displays to show connection within each sub-group type • Data comparison: identify patterns, themes, relationships, and major variables within and between sub-groups • Conclusion drawing and verification: creative and critical analysis of data, acknowledging commonalities and differences, and including any justifiable generalisations • Production of integrative summation
(5) Presentation	To capture the depth and breadth of the topic and produce a comprehensive understanding	• Summary should contribute to a new understanding • Specify implications for practice, research, and policy • Note limitations of the review as a whole

### Variables of interest

The purpose of the review is to gain a comprehensive understanding of the complex and potentially multifaceted relationship between physiological development and health-related behaviours in adolescence. The main variables of interest are theories and hypotheses related to physiological development in adolescence and relationships with health-related behaviour. In particular, the review will aim to assess the strength of such evidence.

### Focus and boundaries

To assist with the classification of the review studies, all identified material will be screened for relevance using the broad inclusion criteria of adolescent physiological development and health behaviours. Physiological development will encompass a broad range of biological systems (e.g. musculo-skeletal, nervous, endocrine, integumentary, cardiovascular, respiratory, digestive, reproductive) and associated biochemical and hormonal processes.

Health-related behaviours will include areas such as diet and nutrition, physical activity, substance use (including smoking), sexual behaviours, and sleep. Outcome measures will potentially refer to any of these systems or behaviours.

Where there is insufficient evidence in the title and abstract to make a decision, full-text papers/reports will be retrieved and narrow screening criteria used on all full text papers.

Narrow screening will focus on the study design, population, intervention, and outcome (SPIO) criteria:

#### Study design

This review will consider all research designs and types of publication. Randomised controlled trials, non-randomised controlled trials, comparative studies, case control studies, and cohort studies will all be considered for inclusion. Qualitative data will be included, if relevant, as will published reports and grey literature such as unpublished dissertations/theses (where accessible) to reduce the likelihood of publication bias.

#### Population

Studies that include adolescents aged nominally between ≥10 and ≤24 years will be included, although no definitive age barriers will be used to avoid excluding potentially relevant research, with the proviso that the topic focus relates to the adolescent life stage. Papers reporting exclusively on younger children and/or adults will be excluded. Studies of non-human subjects will be included if they supplement the knowledge base (e.g. add further information regarding mechanisms).

Studies with an exclusive focus on participants with specific clinical health conditions will be excluded, since these groups may have distinct needs in terms of development and health behaviour. These groups include individuals diagnosed with eating disorders, addictions, or major mental health disorders. Studies that focus exclusively on individuals with morbid (or severe) obesity will be excluded, due to the clinical impact on health and subsequent health-related behaviours.

#### Interventions

All types of intervention will be included, if relating to adolescent physiological development and including explicit or implicit links to health behaviour.

#### Outcomes

Included outcome measures will not be restrictive, with the proviso that there is a relationship between physiological development and health behaviour.

Physiological development will include such elements as relate to healthy and normal functioning and the physical and chemical phenomena involved therein [14].

Health behaviours will relate to behaviours that have the potential to impact on health, such as dietary intake, physical exercise, sexual behaviour, sleep, and substance use (including alcohol, drugs, smoking, and solvents). Papers that solely focus on situations where substance use had evolved into an addiction will be excluded, as will studies involving gambling.

We will include studies that examine pre-adolescent biological development if these studies also relate this to adolescent physiology and health-related behaviour. We will also include studies that assess the impact of adolescent physiological development on health-related behaviour in adulthood.

Theoretical or discussion papers will be included if they contribute to our understanding of mechanisms or effects. We will exclude protocols that do not contain results.

Studies dating from 1980 will be included. Health promotion was formally acknowledged and defined as an important part of healthcare professional’s work by WHO in 1986, as part of the Ottawa Charter [15]. It was therefore largely from this decade onwards that more formal initiatives started to be used and became the focus of research work. However, important earlier theoretical papers which contribute to our understanding will be considered for inclusion.

No initial language limitations will be applied. If necessary, we will seek translation of papers that appears to meet the inclusion criteria. Similarly, no geographical boundaries will be set.

Details regarding inclusion and exclusion criteria are summarised in Table 2.

**Table 2:** Inclusion/exclusion criteria

Inclusion criteria	Exclusion criteria
Studies/reports involving:	Studies/reports involving:
• Adolescents (nominal age range 10–24 years^a^) and • Physiological development, including developmental influencers (e.g. biochemical influences, hormonal activity, enzyme activity, and neurotransmitters), with related outcomes and • Health behaviour (explicit or implicit), including lifestyle or behavioural factors/outcomes, and decision making	• Children (e.g. under 10 years^a^) as sole focus and/or • Adults (e.g. >24 years of age^a^) as sole focus and/or • Atypical or pathological physical/psychological/physiological developmental focus (e.g. eating disorders, addictions, gambling, or major mental health disorders) and their treatment interventions • Sport performance studies • Adolescent pregnancy (as sole focus) • Incidence, prevalence, or trend papers (as sole focus) • Protocols for studies yet to be undertaken

^a^No definitive age barriers will be used to avoid excluding potentially relevant research

The project group for this review will monitor all stages of the review process and is comprised of researchers with a background in public health, neuroscience, adolescence, and systematic reviewing, as well as policy/decision makers with a remit to improve health for young people across Scotland. An advisory group, including lay adolescent members, will provide additional guidance as necessary.

### Literature search

#### Search strategy

A comprehensive search strategy that aims to be both sensitive and specific will be used in this review. An initial search strategy will be devised using the MEDLINE thesaurus and indexing system to identify appropriate MeSH headings and key/text words associated with the terms ‘adolescence’, ‘physiology’, and ‘health-related behaviour’. This will be adapted for use across all included databases as necessary.

#### Stages in the literature search

**Fig. 1:**
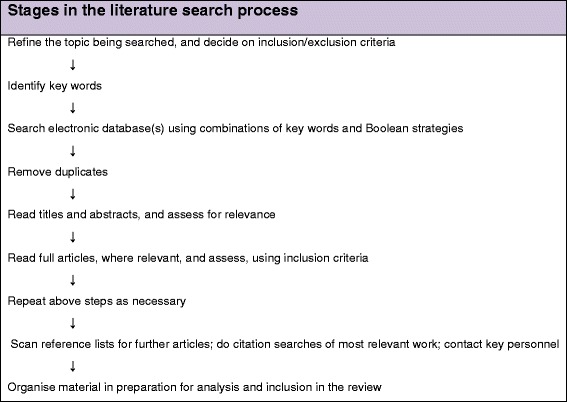
Stages in the literature search process (summarised from Aveyard [18])

Stages in the literature search are summarised in Fig. 1. Although not limited to empirical evidence, we will use the Preferred Reporting of Systematic Reviews and Meta-Analysis (PRISMA) flow chart format to visually display the processes and findings of the review.

#### Databases

Details of databases to be used in the review are given in Table 3.

**Table 3:** Databases to be used in the systematic review

Database	Details
AMED	Allied and Complimentary Medicines Database, with a focus on complimentary medicine, palliative care, and professions allied to medicine
ASSIA	Applied Social Sciences Index and Abstracts; includes literature from psychology, sociology, medicine, anthropology, politics, and law
CENTRAL	Cochrane Central Register of Controlled Trials; summary details of published and unpublished trials
CINAHL	Cumulative Index to Nursing and Allied Health Literature. Includes over 2.6 million records dating back to 1981; books and dissertations also included
EMBASE	Excerpta Medica Database covers biomedical and pharmacological literature
ERIC	Education Resources Information Center (ERIC); described as the world’s largest database of educational literature
HMIC	The Health Management Information Consortium; brings together the bibliographic databases of two UK health and social care management systems: the Dept of Health’s library and information services and King’s Fund information and library services. Includes grey literature
MEDLINE	Covers health-related journals worldwide, focusing on evidence/research based work; includes in-process and non-indexed items
PsycINFO	Abstract database providing systematic coverage of psychological literature as far back as the 1800s
PubMed	Includes Medline, plus a comprehensive and broad-ranging selection of health-related journals and books
Discovery	University of Edinburgh accumulated databases and resources

#### Grey literature

The term ‘grey literature’ came into common use in the 1970s [16] and is generally considered to refer to unpublished research.

In this review, Electronic theses On-line Service (EthOS) 1980–2015 and Zetoc conference proceedings (1980–2015) will be searched electronically. Google and Google Scholar will also be searched using key phrases.

In addition, key professionals and voluntary/third sector organisations will also be contacted for resources and reports that may not have been identified through routine searches of databases.

Reference lists of all eligible research or reports will also be scanned to identify potentially relevant references not retrieved by the database searches.

#### Screening

Two review authors will independently screen potential studies for eligibility at both broad and narrow screening stages. Members of the project or advisory teams will act as third reviewers in the case of eligibility uncertainty or discrepancy.

#### Bibliographic management

A bibliographic data management system (RefWorks™) will be used to store and manage the results of the electronic database searches. This will also provide an audit trail of identified resources and give indicators of how they are subsequently classified and sub-divided during the broad and narrow screening processes.

### Data evaluation

#### Classification of result resources

Data extraction sheets and tables will be developed, tailored to the resources found. These will give an overview summary to assist in the production of groupings and classifications of the types of resources identified by the literature searches.

The different types of study or report will be classified into major groups and then divided into sub-groups, as necessary and relevant.

#### Critical appraisal

To assess the quality of papers selected for inclusion in the review, the UK Critical Appraisal Skills Programme [17] provides tools suitable for the evaluation of individual study quality. Quality assessment will address issues such as whether study aims are clearly stated and whether findings and conclusions are valid and/or credible. Two review authors will independently quality appraise included studies and agree the final assessment; a third reviewer will assist in the event of a discrepancy.

The critical appraisal of studies will assist with assessing the strength of evidence from individual and grouped studies.

### Data synthesis

Once the final number of studies has been identified and appraised, the task of data synthesis will commence. It is anticipated that this will involve analysis within and across groupings, which could potentially include and compare:

- Population/age groupings
- Behaviours and risk
- Developmental stage
- Socioeconomic factors
- Gender
- Individual or multiple lifestyle factors
- Outcome measures
- Theory or hypotheses bases

Depending on the findings, the type and scope of further analyses (e.g. meta-analysis, meta-synthesis, meta-summary) will be decided upon, and sub-set analyses carried out as necessary and/or feasible. Relevant statistical software will be used, if appropriate.

Charts and other visual displays will be used to show the patterns, connections, and variables within and across the data sources. These will assist in the production of an integrative summation, giving a critical analysis of the results as a whole and providing any conclusions, generalisations, and recommendations from the review.

### Presentation

The integrative summation will form the basis of a report on the findings of the review. Presentation and dissemination of results will be through local, national, and international publications and conferences, via a variety of media (e.g. slide presentations, podcasts, written summaries) and for a variety of audiences (e.g. academic/health professionals, lay audiences, including adolescents and pictorial versions for those with reading difficulties).

## Discussion

### Strengths and limitations of the review

This review seeks to provide a comprehensive understanding of the complexities surrounding adolescent physiological development and the relationship and links to health behaviour. As such, the review aims are ambitious in their scope and depth. Although the review will use a clear and systematic approach to searching, screening, and reviewing studies, the search strategies and engines may not capture all relevant material. The findings may also be open to selection bias. The presence of the wider project research team and advisory/reference groups will serve to minimise such bias.

### Relevance of the review

It is anticipated that the results of the review will be of interest to a wide variety of policy makers, organisations, and individuals working with adolescents across health, social care, and education. We will therefore seek to make the findings from the review widely available. The findings will also be used to inform future research strategies, by identifying areas where further evidence would be beneficial and providing the necessary background information to justify research funding allocation and priority.

## Additional file

Additional file 1:
**Adolescent project overview.** This provides details of where this current review is placed within the broader context of adolescent study by the project team. (DOCX 28 kb)
