# Supplementary material for: A systematic review of adolescent physiological development and its relationship with health-related behaviour: a protocol
Source: Syst Rev. 2016 Jan 5;5:3. doi: 10.1186/s13643-015-0173-5 (PMC4700763; doi:10.1186/s13643-015-0173-5)
Supplement: Additional file 1: — Adolescent project overview. This provides details of where this current review is placed within the broader context of adolescent study by the project team. (DOCX 28 kb) [file 13643_2015_173_MOESM1_ESM.docx]

**Additional file 1 Adolescent project overview**

| **MICRO ENVIRONMENT (INDIVIDUAL OR PROXIMAL DETERMINANTS)** | | **MACRO ENVIRONMENT**  **(WIDER or DISTAL DETERMINANTS)** | | | |
| --- | --- | --- | --- | --- | --- |
| **PHYSIOLOGY/BIOLOGY**  E.g. Puberty, Neurobiology, Endocrinology, Immunology | **PSYCHOLOGICAL**  E.g. cognition, autonomy, resilience | **ENVIRONMENT: PHYSICAL**  E.g. Housing, Transport, Green spaces, safety | **ENVIRONMENT: SOCIAL**  E.g. family, peers, school, community | **ENVIRONMENT: SOCIO-ECONOMIC**  E.g. Income, Occupation, Education, Poverty | **ENVIRONMENT: CULTURAL**  E.g. Social norms/attitudes, Media |
| **Phase 1** | **Phase 2** | **Phase 3** | | **Phase 4** | |

Stage 5. Cross-synthesis of all evidence obtained from all phases

Stage 4. Dissemination, and incorporation of evidence into policy and practice

Health related behaviours could include: tobacco use, alcohol and drug misuse, sexual risk taking, physical activity, healthy eating, sleep either alone or in combination

Stage 3: Cross-study synthesis of the evidence to identify commonalities and time-points where interventions may be most effective.

Stage 2: Search and synthesise empirical evidence that uses the theories to explain or predict health related behaviours (cohort studies in humans).

Stage 1: Identify candidate theories (can be from animal models)
